# Marine Microbial-Derived Molecules and Their Potential Use in Cosmeceutical and Cosmetic Products

**DOI:** 10.3390/md15040118

**Published:** 2017-04-12

**Authors:** Cinzia Corinaldesi, Giulio Barone, Francesca Marcellini, Antonio Dell’Anno, Roberto Danovaro

**Affiliations:** 1 Department of Sciences and Engineering of Materials, Environment and Urbanistics, Università Politecnica delle Marche, 60131 Ancona, Italy; 2 Department of Life and Environmental Science, Università Politecnica delle Marche, 60131 Ancona, Italy; g.barone@pm.univpm.it (G.B.); a.dellanno@univpm.it (A.D.); 3 Ecoreach Ltd., 60131 Ancona, Italy; f.marcellini@univpm.it; 4 Stazione Zoologica Anthon Dohrn, 80121 Naples, Italy; r.danovaro@univpm.it

**Keywords:** marine bacteria, marine fungi, cosmetics and cosmeceuticals, marine bioactive compounds

## Abstract

The oceans encompass a wide range of habitats and environmental conditions, which host a huge microbial biodiversity. The unique characteristics of several marine systems have driven a variety of biological adaptations, leading to the production of a large spectrum of bioactive molecules. Fungi, fungi-like protists (such as thraustochytrids) and bacteria are among the marine organisms with the highest potential of producing bioactive compounds, which can be exploited for several commercial purposes, including cosmetic and cosmeceutical ones. Mycosporines and mycosporine-like amino acids, carotenoids, exopolysaccharides, fatty acids, chitosan and other compounds from these microorganisms might represent a sustainable, low-cost and fast-production alternative to other natural molecules used in photo-protective, anti-aging and skin-whitening products for face, body and hair care. Here, we review the existing knowledge of these compounds produced by marine microorganisms, highlighting the marine habitats where such compounds are preferentially produced and their potential application in cosmetic and cosmeceutical fields.

## 1. Introduction

The oceans host a huge biodiversity, with more 250,000 species described and up to 8.5 million species still to be discovered [1], but estimates on microbial diversity are largely unknown [2]. In recent decades, the exploration of the oceans has allowed the discovery of a multitude of previously unknown habitats characterized by extreme conditions [3]. These environments host a variety of organisms adapted to these conditions and producing a wide range of active biomolecules [4,5]. More than 25,000 new biologically active compounds have been identified in the past fifty years, with an increment of 5% per year and 1378 new molecules identified in 2014 alone [6]. Among marine organisms, microorganisms, including fungi, fungi-like protists (such as thraustochytrids) and bacteria, have attracted great attention as potential leading compound producers [6,7,8].

**Figure 1:**
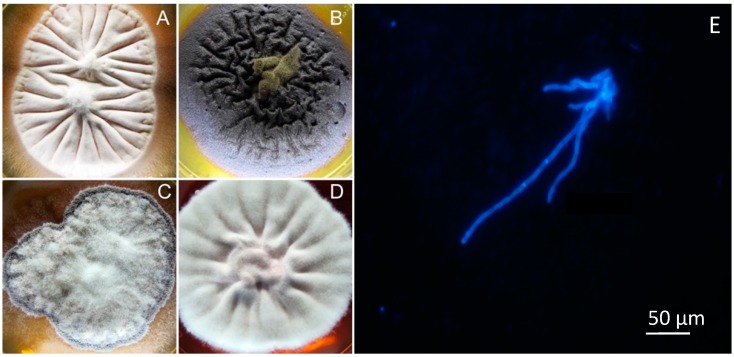
Marine Fungi, *Penicillium* sp. (**A**,**C**); *Cladosporium* sp. (**B**); *Aspergillus* sp. (**D**) and fungal hyphae in marine sediment samples stained with Calcofluor (**E**).

Fungi are abundant and ecologically relevant members of marine microbial assemblages (Figure 1). They were believed to be rare in marine environments, but recent studies based on molecular and metagenomics approaches have revealed an unexpected diversity from coastal to deep-sea ecosystems [9,10,11]. Deep-sea fungi have been less described in terms of their abundance, diversity and ecological role, but are potentially important and productive sources of bioactive molecules [12].

**Figure 2:**
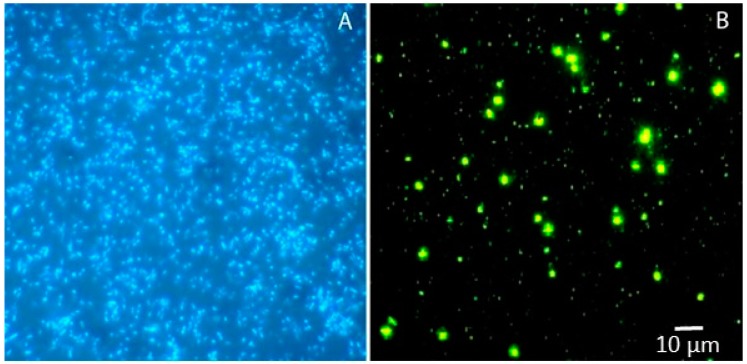
Marine bacteria in seawater samples stained with DAPI (**A**) and SYBR Green I (**B**).

Bacteria are typically the most abundant (ca. 10^29^ cells, Figure 2) and diverse members of the microbial assemblages in the oceans, where they are key players in biogeochemical processes and fluxes of energy and matter [13]. Several bacterial species are distributed across all marine ecosystems worldwide, and are currently exploited for a number of biotechnological applications. 

A multiplicity of compounds from marine and marine bacteria and fungi such as polyketides, alkaloids, peptides, proteins, lipids, mycosporines and mycosporine-like amino acids, glycosides, isoprenoids and hybrids have great potential in cosmeceutical and cosmetics since they exhibit photo-protective, anti-aging, anti-microbial, anti-oxidant and moisturizing activities [7,14]. Such compounds show specific chemical structures and activities up to two orders of magnitudes higher than those reported by species inhabiting terrestrial systems [8,15].

The global market for cosmetic and cosmeceutical products is forecasted to grow at a rate of 4.3% by 2022 with a value of USD 430 billion (https://www.alliedmarketresearch.com). Photo-protective, skin-care and hair-care products drive this trend of increasing demand. In addition, consumers’ demand is turning to natural products given health concerns and popular trends, forcing research to discover new compounds from the abundant and alternative source represented by marine organisms [16].

Bioactive compounds produced by marine microbes are still largely unexplored and unexploited [2]. Therefore, the identification of marine microbial-derived molecules for (bio)technological and industrial purposes has huge potential for new discoveries [7]. Since bioactive compounds from marine photosynthetic microorganisms (e.g., cyanobacteria and microalgae) have been extensively reported in literature, the present review provides an overview of the different bioactive compounds produced from marine and marine heterotrophic bacteria, fungi and fungi-like protists such as thraustochytrids, and their potential applications in the cosmeceutical and cosmetic industry. We also highlight the untapped potential of these microorganisms as source of photo-protective, moisturizing, anti-wrinkle, skin-whitening products and other commonly active ingredients and/or adjuvants included in the composition of personal care products (i.e., anti-oxidant, anti-microbial and preservatives).

## 2. Photo-Protective Compounds

There is evidence that prolonged human exposure to UVA (320–400 nm) and UVB (280–320 nm) radiation may result in acute and/or chronic effects on the skin and on overall human health [17,18]. Growing awareness of the risks associated with skin exposure to UV radiation over recent decades has led to increased production and consumption of solar products worldwide, reaching unexpected levels [19].

Several marine organisms have evolved a set of mechanisms to protect themselves from the harmful effects of UV radiation, producing UV-absorbing compounds such as scytonemins (exclusively in cyanobacteria), mycosporines, mycosporine-like amino acids (MAAs), carotenoids and melanin [20,21]. These compounds offer a great potential for the development of novel UV filters to be included in sunscreen products. Generally, common sunscreen products contain organic and/or inorganic filters [22]. However, recent investigations have proved that traditional UV-filters and other ingredients (e.g., preservatives) might have harmful effects not only on human skin but also on marine life [19,23]. This evidence has stimulated new research on alternative and possibly eco-friendly photo-protective compounds. Marine organisms are a reliable source of photo-protective compounds. In particular, photosynthetic organisms have been thoroughly investigated as sources of several compounds including mycosporines, mycosporine-like amino acids and several other UV filters such as carotenoids and scytonemin [24,25]. Despite the large contribution of heterotrophic microorganisms to marine biodiversity and biomass, the identification of UV filters produced by these microbial components has received much less attention. Here, we report the main bacterial and fungal-derived photo-protective compounds, so far studied (Table 1).

### 2.1. Mycosporine and Mycosporine-Like Amino Acids

Mycosporines and mycosporine-like amino acids (MAAs) are low molecular weight water-soluble molecules with great application in several fields of cosmetic and cosmeceutical industries. These compounds are synthesized and accumulated by a wide range of organisms such as cyanobacteria, prokaryotes and fungi andalgae, whereas other marine organisms (metazoans) obtain MAAs from their feed [20,90]. Available evidence suggests that these molecules are not exclusively involved in photoprotection, but can have a role in thermal, salt and desiccation stress while in fungi are involved in sporulation and germination processes [91,92,93]. Mycosporines are composed of either an aminocyclohexenone or an aminocycloheximine ring with nitrogen or imino alcohol substituents and absorb in the range of 310–320 nm [93,94]. Mycosporine-like amino acids (MAAs) are imine derivatives of mycosporines which contain an amino-cyclohexenimine ring linked to an amino acid, amino alcohol or amino group with absorption in the range of 320–360 nm [20]. MAAs are favored to mycosporines as photo-protective due to their wide spectrum of absorbance and the ability to dissipate UV radiation without producing reactive oxygen species (ROS) [20,95,96].

Previous investigations have revealed that fungal strains isolated from hypersaline waters and polar glacial ice are able to synthesize mycosporines, as well as unidentified yet UV-absorbing compounds (possibly MAAs, [26]; Table 1). In particular, mycosporine–glutaminol–glucoside and mycosporine–glutamicol–glucoside were detected in black yeasts *Phaeotheca triangularis*, *Trimmatostroma salinum*, *Hortaea werneckii* and *Aureobasidium pullulans*, as well in a basidiomycetous yeast, the *Cryptococcus liquefaciens* [26]. Despite previous studies revealing that bacteria might be able to synthesize MAAs, available information for these microorganisms is very limited. MAAs have been found in microorganisms including *Pseudonocardia* sp. strain P1 (Actinomycetales) and *Micrococcus p. AK-334*, whereas in other bacteria such as *Actinosynnema mirum* DSM 43827 only genes involved in MAAs biosynthesis have been identified [27]. These biosynthetic gene clusters were also expressed in engineered hosts (i.e., *Streptomyces avermitilis* SUKA22), which were able to accumulate different types of MAAs including shinorine (mycosporine-glycine-serine) and porphyra-334 (mycosporine-glycine-threonine) and a novel MAA [27].

The potential for cosmetics of mycosporines and MAAs, especially extracted from microalgae, is well known [25,91,97,98,99,100] and proved by several patents. However, only very few UV-screening and anti-aging products containing mycosporines and MAAs are commercially available (such as the MAA produced by the red alga *Porphyra umbilicalis*) [24,25,101], and to our knowledge no cosmetic containing such compounds from fungi and bacteria has been developed so far. Diverse synthetic analogues of MAAs (including analogues of mycosporine-glycine) have been tested for commercial purposes but most of them were not sufficiently stable for commercial application as sunscreen products [101].

### 2.2. Carotenoids

Carotenoids are the most common pigments in nature [102] and have several applications as colorants, food supplements and cosmetics/nutraceuticals; they are also used for medical and biotechnological purposes [103]. More than 750 carotenoids have been described, but lycopene, β-carotene, astaxanthin, zeaxanthin and lutein are the most important from a commercial point of view [28]. These pigments have diverse biological functions, therefore fit into a wide range of cosmetic and cosmeceutical applications [22,28]. Marine carotenoids have significant anti-oxidant and anti-inflammatory effects and may contribute to skin photo-protection and inhibit adverse processes induced or mediated by solar UV radiation. It has been suggested, indeed, that routine consumption or topical treatment of carotenoids such as lycopene, β-carotene and lutein may provide efficient protection against the harmful effects of solar UV radiation [101]. 

**Figure 3:**
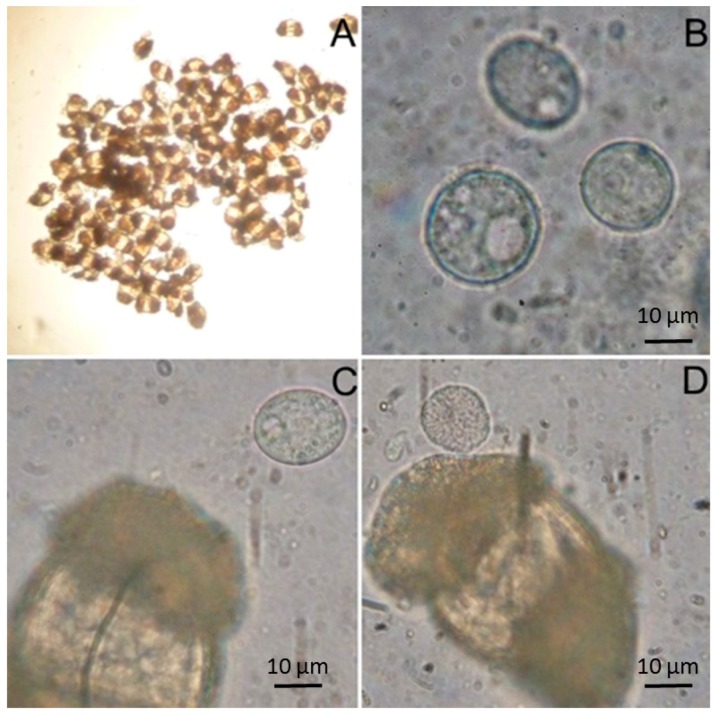
Marine thraustrochytrids associated with pollen grains (**A**,**C**,**D**) and free (**B**).

Despite carotenoids being photo-protective compounds, they are more used for their anti-oxidant properties in sunscreen formulations [22,104,105]. Besides photosynthetic organisms, heterotrophic bacteria and marine fungi (especially pigmented yeasts), thraustochytrids (generally defined as fungi-like protists) are also a relevant source of carotenoids [28,29,30,33]. However, these microorganisms have not been examined as extensively as the photosynthetic organisms (i.e., algae) for the production of carotenoids [106]. Among marine heterotrophic microorganisms, bacteria such as the genera *Paracoccus* and *Agrobacterium* have been reported as promising astaxanthin producers [28,31,32] (Table 1). Astaxanthin is also produced by several yeast species belonging to the genera *Rhodotorula, Phaffia, Xanthophyllomyces* [32]. Although the production from yeasts and bacteria is lower compared to algae, yeasts have higher growth rates and easier cultivation conditions [29,107]. Thraustochytrids have a wide geographical distribution from the polar to tropical regions, and they include planktonic and benthonic forms inhabiting various habitats such as sediments of mangroves, estuaries and deep-sea ecosystems (Figure 3, [34]). These fungi-like protists such as *Thraustochytrium* strains ONC-T18 and CHN-1, *Thraustochytriidae* sp. *AS4-A1* (*Ulkenia* sp.) and *Aurantiochytrium* sp. KH105 synthesize different carotenoids including β-carotene, astaxanthin, zeaxanthin, cantaxanthin, phoenicoxanthin and echinenone [33]. Engineering approaches have allowed the increase in production of carotenoids (even nine-fold increased astaxanthin content production) such as in *Aurantiochytrium* sp. SK4 [35]. From this perspective, the development of genetic tools and genome sequencing of thraustochytrids are fundamental to expand our knowledge of these promising sources of carotenoids to be employed in cosmetic products.

### 2.3. Benzodiazepine Alkaloids

Benzodiazepine alkaloids, e.g., circumdatins A–H, are widespread compounds produced by terrestrial and marine fungi. Circumdatin I, C and G have been isolated from the mycelium of a marine fungus of the genus *Exophiala*. These compounds showed high UV-A screening activity, exhibiting ED50 (i.e., effective dose for 50% of the tests) values of 98, 101 and 105 μM, and were more efficient of oxybenzone (ED50, 350 μM) than is currently used sunscreen filter [36].

## 3. Anti-Aging Products

Skin aging involves changes in skin physical properties creating visible signs on the skin surface due to the degradation of the extracellular matrix in both the epidermal and dermal layers. Anti-aging products are among the most marketed cosmetics/cosmeceuticals worldwide and the global anti-aging market is expected to reach USD 216.52 billion in 2021, growing at 7.5% (CAGR) from 2016 to 2021 (www.zionmarketresearch.com). Such personal care products, including face, hair and body treatments are widely used to contrast cutaneous dryness, roughness, the depth of wrinkles and loss of skin tone. Generally, all anti-aging formulations contain moisturizing substances. The maintenance of hydration, indeed, is pivotal for keeping skin functions. The external application of lipid compounds that have the ability to limit water loss or molecules that produce bonds with water may have the potential of mimicking the natural hydrating mechanisms of the skin. Among these substances, marine organisms produce several high molecular weight molecules such as polysaccharides, fatty acids (PUFA, sophorolipids, rhamnolipids and mannosylerythritol) and proteins (collagene) that are widely used in skin care (facial care, facial cleansing, body care, baby care) for their softening and smoothening effects on the skin. Several bioactive substances with anti-wrinkling action of marine origin are already produced on a large scale. Among these, exopolysaccharides (EPSs) and fatty acids are of great importance for anti-aging products (Table 1).

### 3.1. Exopolysaccharides 

Among the bioactive substances with anti-wrinkling action of marine origin, polysaccharides of microbial origins, especially EPSs, are the most used. EPSs are high molecular weight carbohydrate polymers that in nature are involved in a variety of mechanisms, from attachment to intra- and inter-specific communication and competition [37]. EPSs are produced not only by bacteria but also by other microorganisms such as fungi and microalgae. However, bacteria are amenable to the largest production [38]. EPSs constitute a class of products with properties including emulsifying, thickening, absorption and gel formation [39,40]. 

In recent years, there has been a growing interest in isolating new EPSs particularly from extreme environments such as deep-sea hydrothermal vents, cold seeps, polar and hypersaline ecosystems [37,41,42,43]. Among the most important producers of EPS there are several taxa of bacteria and molds including *Agrobacterium* sp., *Alcaligenes faecalis*, *Xanthomonas campestris*, *Bacillus* sp., *Zymonas mobilis* and *Aureobasidium pullulans* [44] (Table 1). 

EPSs (HYD657) secreted by the marine bacterium *Alteromonas macleodii* have already found application in cosmetics and are commercially available [16]. Similarly, a mixture of EPSs from *Pseudoalteromonas* sp. isolated from Antarctic waters is included in the formulation of anti-aging products. This mixture, obtained through fermentation, enhances the synthesis of collagen I, contributing to the amelioration of skin structural properties [16]. Other anti-aging products containing EPSs include those based on extracts from marine microbes *Pseudoalteromonas antarctica* and *Halomonas eurihalina*. Recently, the *Vibrio diabolicus*, a deep-vent marine bacterium, has been discovered to produce an exo saccharide (HE 800) structurally analogous to hyaluronic acid with unique functions that stimulate collagen structuring [41].

### 3.2. Fatty Acids

Fatty acids are known not only as dietary supplements, but they also have a broad spectrum of topical applications in cosmetics and cosmeceuticals thanks to their role in soft tissue repair and skin nourishment through stimulation of collagen production as well as anti-inflammatory and wound healing [108]. Among the different fatty acids, polyunsaturated fatty acids (PUFA), and specifically the omega-3 fatty acids docosahexaenoic acid (DHA) and eicosapentaenoic acid (EPA) have been linked to several health benefits [109]. Most marine animals obtain long-chain PUFAs from their diets (i.e., as products of photosynthetic processes) and few are known to produce these compounds de novo (microalgae, bacteria, thraustochytrids and fungi). The main source of omega-3 fatty acids for human consumption is wild fish [45,46,110]. However, due to its decline and the consequent increase in price, to satisfy current demand of DHA and EPA research has started to focus on alternatives to fish oil such as oil from plants, algae, bacteria and fungi. The term “single-cell oils” (SCOs) refers to oils produced by single-cell microorganisms such as yeasts and molds [47]. SCOs produced by microorganisms offer many advantages compared to fish oil, including the higher growth rate and oil content and the presence of a number of natural anti-oxidants such as carotenoids that prevent omega-3 fatty acids oxidation [48]. Among marine microorganisms, thraustochytrids, fungi and bacteria have received lower attention although have great potential to produce these fatty acids. Thraustochytrids have been isolated from marine environments through the classic technique of the pine pollen grains as bait (Figure 3) [34], and quantified in the world’s oceans [10]. Since the 1990s they have been used for the industrial production of DHA due to their high production per unit of biomass and fast growth rate [49,50,51,52,111]. These microorganisms can accumulate more than 50% of their weight as lipid drops, with a concentration of DHA higher than 25% of the total lipids. The lipids of thraustochytrids contain specifically eicosapentaenoic acid (EPA), docosapentaenoic (DPA) and docosahexaenoic acid (DHA) and have a higher level of oxidative stability than that of fish oil. The development of refined techniques has been important for the cultivation, isolation and identification of thraustochytrids for industrial purposes. In particular, there is evidence that species belonging to *Schizochytrium*, *Aurantiochytrium* and *Ulkenia* isolated from several marine ecosystems, including sandy beaches and mangrove forests, are the major producers of DHA [52]. DHA-rich oils from thraustochytrids are currently on the market particularly for applications in nutraceuticals and aquaculture [33]. However, they also have a great potential for cosmetic and cosmeceutical applications.

Also, yeast species isolated from seawater (e.g., *Rhodotorula mucilaginosa* AMCQ8A) are capable of producing high biomass with high lipid yield [53,54]. Similarly, several oleaginous marine bacteria have been reported to produce important PUFAs (Table 1), such as the marine *Moritella dasanensis* [55]. PUFA-producing bacterial isolates are known to be associated with high-pressure, low-temperature, deep-sea habitats. In literature, species belonging to *Shewanella* and *Colwellia* genera have been reported to produce DHA and EPA [56]. However, to our knowledge, their use in cosmetic and cosmeceutical sectors has not been addressed yet.

### 3.3. Antioxidant Compounds

Antioxidant compounds are added to prevent oxidation of ingredients in cosmetic formulations. These compounds also have a fundamental role in protecting the skin from oxidation induced by reactive oxygen species (ROSs) due to natural oxidation occurring within the cells, stimulated by UV radiation and loss of skin moisture. At present, several synthetic anti-oxidants have been used in cosmetic and cosmeceutical products such as butylated hydroxyanisole, butylated hydroxytoluene, tertiary butyl hydroquinone and propyl gallate [31]. Since synthetic compounds might be toxic [79], natural anti-oxidants have been investigated to be used in cosmetics. Marine anti-oxidants include mycosporines, MAAs, carotenoids and other compounds that may serve multiple functions within cosmeceutical formulation [28,29,57,100].

MAAs may protect the skin not only against UV radiation but also exhibit a high anti-oxidant activity, scavenging superoxide anions and inhibiting lipid peroxidation [57,58,59,95]. The properties of MAAs as UV screens and ROS scavengers suggest that they could be used in sunscreen products [96]. Their roles as UV-absorbing and anti-oxidant compounds in human fibroblast cells have been rarely investigated [58]. However, previous studies revealed that mycosporine-glycine has strong anti-oxidant, anti-inflammatory and anti-aging activity providing new insights into the application of MAAs in the cosmetic/cosmeceutical sectors.

Carotenoids are known for their powerful anti-oxidant activity [112,113]. Astaxanthin is among the strongest anti-oxidants due to its structure and better biological activity than other anti-oxidants [60,113,114]. In addition, thanks to the discovery of new species, novel and rare carotenoids are being screened [87]. Two rare carotenoids with relevant anti-oxidant action (i.e., saproxanthin and myxol) have been isolated from new strains of marine bacteria belonging to the family Flavobacteriaceae [61] (Table 1). Saproxanthin or myxol addition to cosmetics might help to reinforce biological membranes, decreasing permeability to oxygen and enhancing protection against oxidation. The anti-oxidant activities of saproxanthin and myxol were even greater than those of the commonly used zeaxanthin and β-carotene [61]. However, these new and rare marine carotenoids require a thorough evaluation before their implementation within cosmeceutical products [105].

Marine fungi are an excellent source of anti-oxidant compounds. The marine fungus *Acremonium* sp., was found to produce four novel hydroquinone derivatives with significant anti-oxidant activity [62]. Similarly, the *Epicoccum* sp. isolated from the alga *Fucus vesiculosus* was found to produce an isobenzofuranone derivative (4,5,6-trihydroxy-7-methylphthalide) with high a,a-diphenyl-picrylhydrazyl (DPPH) radical scavenging effects [64]. Recently, eight secondary anti-oxidant metabolites were identified in *Aspergillus wentii* EN-48 isolated from brown algae [63]. The activities of these anti-oxidants were found to be considerably higher than the synthetic ones commonly used such as butylated hydroxytoluene [63]. Another interesting anti-oxidant is represented by the exopolysaccharide EPS2 isolated from the marine filamentous fungus *Keissleriella* sp. YS 4108 that displayed profound scavenging activities of superoxide radicals [65]. Other anti-oxidant compounds arediketopiperazine alkaloid, golmaenone and related alkaloid, as well as dihydroxy isoechinulin A and related echinulin, which have been isolated from the culture broth of the marine fungus *Aspergillus* sp. [66,67]. Golmaenone and related alkaloid exhibited a significant radical scavenging activity against 1,1-diphenyl-2-picrylhydrazyl (DPPH) with IC_50_ (i.e., the concentration which shows 50% inhibition) values of 20 μM, similarly to ascorbic acid (IC_50_, 20 μM). Furthermore, these compounds also displayed UV-A screening function with ED_50_ values of 90 and 170 μM, thus were more efficient than oxybenzone (ED_50_, 350 μM) [66].

## 4. Skin-Whitening Products

Public interest in skin-whitening cosmetics is increasing notably and this market is forecasted to reach USD 23 billion by 2020 [115]. Skin whitening refers to the use of natural or synthetic substances that provide an even pigmentation by reducing the melanin concentration in the skin. This practice may be driven by dermatological needs such as skin hyperpigmentation due to autoimmune conditions, exposure to UV radiation, genetic factors and hormonal changes that can induce overproduction of melanin in the skin [116]. Nowadays, skin whitening is more often practiced for aesthetic ends for a whiter and paler skin tone, as it is synonymous with youth, whereas darker skin is associated with lower social classes [117]. 

Melanin biosynthesis can be reduced by the inhibition of the tyrosinase enzyme, the inhibition of melanocytes’ metabolism and proliferation [118]. Numerous natural compounds from marine organisms (e.g., hydroquinones, kojic acid, azelaic acid and electron-rich phenols) have already been employed as skin whiteners and in particular as tyrosinase inhibitors, although several of these have been proven to have negative effects on human health [119,120]. Several compounds, including those from marine and marine bacteria and fungi, have been investigated for their employment in cosmetic products [116]. However, few microbial taxa have been investigated for the production of these inhibitors [121]. In recent years, research focused on marine microorganisms producing skin-whitening compounds such as kojic acid, methylene chloride, azelaic acid and others.

Kojic acid (5-hydroxy-2-(hydroxymethyl)-4H-pyran-4-one) is a water-soluble fungal secondary metabolite produced by *Aspergillus* and *Penicillium* species (Table 1). Other marine and marine fungi have been observed to produce such a compound, which owns anti-oxidant, anti-microbial and anti-inflammatory properties, and have significant tyrosinase inhibiting activity [68,122,123]. Two derivatives of kojic acid, kojic acid dimethyl ether and kojic acid monomethyl ether, as well as phomaligol A, were identified from broth of marine fungi *Alternaria* sp. isolated from marine green algae with tyrosinase inhibitory activity [69]. Similarly, azelaic acid (1,7-heptanedicarboxylic acid) is a reliable inhibitor of tyrosinase [74,75,124] produced by the fungus *Malassezia* sp., which inhabits almost every habitat in the marine environment [76]. However, there is no direct evidence on the industrial potential of these marine fungi as source of azelaic acid. Also, the fungal strain H1-7 of *Trichoderma* sp. has been found to produce tyrosinase inhibitors [125]. A competitive inhibitor of tyrosinase (5.4 × 10^5^ U·mL^−1^) similar to the structure of homothallin II was isolated from *T. viridae* strain H1-7 from marine sediments. It inhibited the enzyme by binding to the copper active site. In addition, *T. viridae* strain H1-7 produced seven different melanogenesis inhibitors, but not all of them showed inhibition of tyrosinase [73]. The marine fungus *Botrytis* sp., isolated from the surface of the marine red alga *Hyalosiphonia caespitosem*, was found to produce an α-Pyrone derivate (6-[(E)-Hept-1-enyl]-α-pyrone) characterized by anti-tyrosinase activity (IC_50_ = 4.5 μM) [70]. Two compounds, 6-*n*-pentyl-α-pyrone and myrothenone A, from marine fungus *Myrothecium* sp. MFA 58, exhibited stronger activity than kojic acid (IC_50_ = 7.7 μM), with IC_50_ values of 0.8 and 6.6 μM, respectively [71]. Sesquiterpenes (i.e., 1β,5α,6α,14-tetraacetoxy-9α-benzoyloxy-7β H-eudesman-2β,11-diol and 4α,5α-diacetoxy-9α-benzoyloxy-7βH-eudesman-1β,2β,11, 14-tetraol) with tyrosinase inhibitory activities were isolated from the marine fungus *Pestalotiopsis* sp. Z233. [77]. Other compounds such as 2,4-dihydroxybenzoic acid, caffeic acid, benzene-acetic acid-α,4-dihydroxy, benzeneacetic acid-2-hydroxy, benzenepropanoic acid-α-hydroxy were isolated from the mycelia of *Aspergillus unguis* SPMD-EGY [126]. A recent patent based on chrysophanol as skin whitening extracted from the marine fungus *Microsporum* sp. was also developed (U.S. patent 20140056834A1).

So far, bacteria have been relatively less studied for their potential role in the production of skin-whitening compounds. However, a novel species of the marine bacteria *Pseudomonas* was found to produce methylene chloride, which reduced the pigmentation of human melanocytes and cultured skin cells by inhibiting the expression of tyrosinase [78]. In addition, tyrosinase inhibitors were reported from the marine bacterium *Thalassotalea* sp. PP2-459 isolated from a bivalve. The tyrosinase inhibitors identified as thalassotalic acid A, B and C, with IC_50_ values of 130, 470 and 280 μM, respectively. Thalassotalic acids are *N*-acyl dehydrotyrosine derivatives produced by this bacterium, thalassotalic acid A being comparable to the inhibitory activity of arbutin and could be used as a whitening agent or in preventing browning of foods. They suggest that the presence of a carboxylic acid and a straight aliphatic chain increased enzyme inhibition within this structural class of inhibitors [72].

In addition, the ketocarotenoid astaxanthin owns interesting depigmentation properties. There is evidence, indeed, that astaxanthin, which is also produced by marine yeasts and other taxa, can reduce melanin production by 40% in skin cells protecting skin from flakes and age spots [32]. To our knowledge, skin-whitening compounds used in cosmetic products are mostly extracted from terrestrial organisms although the huge number of marine skin whitening molecules offers new opportunities for the cosmetic market.

## 5. Additives and Other Active Ingredients of Cosmetic Products

Cosmetic and cosmeceutical products include, besides active ingredients (as described above), excipients and additives such as thickening agents, stabilizers, preservatives, colorants and perfumes. While the active ingredients, such as photo-protective compounds, are the main compounds that determine the function of the products, excipients have the purpose of dissolving the active compound in other ingredients. They regulate the delivery of the active ingredients as well as the aesthetical presentation of the product. Stabilizers maintain the stability of the cosmetic product during its lifetime and thickeners increase the viscosity of the product maintaining a proper texture of the cosmeceutical product, which is needed to distribute the active ingredients. 

### 5.1. Antimicrobial Compounds and Preservatives

Preservatives are added to cosmetic products to prevent or delay their alteration and to protect them from microbial contamination. 4-hydroxybenzoate alkyl esters, called parabens, have been largely used as preservatives in food and cosmetic industries. The marine bacterial strain, A4B-17, belonging to the genus *Microbulbifer* isolated from an ascidian, was found to produce 4HBA and its esters. Such compounds were effective in preventing the growth of yeasts, molds and gram-positive bacteria [81]. Among anti-microbial compounds of marine origin, chitosan is widely used in cosmetics and cosmeceuticals. Chitosan is a polysaccharide of basic nature made mostly of glucosamine and a variable number of GlcNAc residues bound through β-1,4-linkages obtained from partial deacetylation of chitin [79]. Chitin is among the most abundant natural polysaccharides [127]. This polymer characterizes the exoskeleton of marine arthropods or the cell walls of fungi [121,122,123].

Chitosan shows anti-microbial activity against bacteria, viruses and fungi [80]. In addition to anti-microbial activity, chitosan and derivatives have several beneficial properties and have numerous applications in cosmeceuticals [128,129]. Unlike other disinfectants, chitosan has a higher anti-microbial and broader spectrum activity and lower toxicity towards humans. However, the actual mechanism of chitosan is not yet fully understood [130], and possible action mechanisms of chitosan and its derivatives have been proposed. Low molecular weight chitosan could penetrate cell walls of bacteria and then combine with DNA-inhibiting transcription [80,131,132]. High molecular weight chitosan could, instead, interact with cell surfaces and consequently alter cell permeability, impeding essential solutes transport into the cell [133,134]. Antifungal activity is attributed to the ability of chitosan to form a permeable film at the interface and has two functions: direct interference of fungal growth and activation of several defense processes [135].

Fungi could be a useful source of chitin and chitosan [128,136]. In fact, chitin constitutes 22%–44% of cell walls of fungi depending on the life stage or morphology, and types and amount of polysaccharides change greatly among taxa. Generally, Zygomycetes contain chitin/chitosan, Chytridiomycetes contain chitin/beta-glucan, Ascomycetes contain chitin/mannan/beta-glucan, and Basidiomycetes contain chitin/beta-glucan [137]. Chitin and chitosan from fungi lack proteins that could cause allergy reactions as in the case of crustacean-derived chitosan [80]. In addition, advances in fermentation technology suggest that the cultivation of selected fungi is less expensive and easier compared to other sources, hence reducing time and costs required for the chitosan production.

Carotenoids also have interesting anti-microbial properties [28]. Astaxanthin, for example, is of particular interest for anti-microbial activity, anti-wrinkle and anti-acne effects and can be used in products for skin conditioning to avoid dryness and decrease swelling under the eyes [32].

### 5.2. Surfactants, Emulsifiers, Thickeners, Stabilizers and Moisturising

Surfactants and emulsifiers are amphipathic compounds having both a hydrophilic and a hydrophobic part [138]. These compounds include protein polysaccharide complexes, glycolipids and lipopeptides from a wide range of marine and marine bacteria and fungi. Marine microorganisms including *Acinetobacter*, *Arthrobacter*, *Pseudomonas*, *Halomonas*, *Myroides*, *Corynebacteria*, *Bacillus*, *Alteromonas* sp. have been studied for production of biosurfactants and bioemulsifiers [89] (Table 1). Among these, the family of compounds derived from chitin display properties as emulsifiers because they are good polymer matrices for the delivery of bioactive compounds, especially of hydrophilic nature. Previous investigations provide evidence that chitosan has a greater water-binding capacity compared to methyl cellulose commonly used in cosmetic and cosmeceutical formulations, suggesting the suitability of high molecular weight chitosan as skin moisturizer and as delivery system in cosmeceutical preparations for anti-aging products [82]. Chitosan in fiber or film state, is mainly applied for improving the epithelial layer and increasing granular density of skin [83]. Indeed, it stimulates fibroblast production that in addition to their moisturizing and anti-microbial properties provide them with remarkable healing properties [84]. A chitosan derivative, carboxymethyl chitosan (CMCS), containing active hydroxyl, carboxyl and amine groups is soluble in water at neutral pH [85] and has anionic functionality, high viscosity, large hydrodynamic volumes, cation-binding characteristics, large osmotic pressures and gel-forming capabilities [83]. Due to all these characteristics, chitosan and chitosan derivatives are very attractive candidates for applications as absorption promoters and hydrating agents, anti-microbial and anti-oxidant agents, delivery system and stabilizers [86]. Chitin nanofibrils are also able to associate with other compounds such as vitamins, carotenoids and collagen, facilitating the penetration into the skin [84]. Due to the proprieties of bio-adhesivity, film formation, stiffness and curl retention to synthetic polymers, chitosan is also used as a hair care ingredient for shampoo, hair gel, hair colorants, hair sprays, permanent wave agents, hair colorants, styling lotions, hair sprays and hair tonics [87]. In addition, since some derivatives of chitin and chitosan (e.g., glyceryl chitosan) form foam and have an emulsifying action, they can be used directly in shampoo [138]. Even carotenoids, such as astaxanthin, have application in hair care products to protect hair from sunlight exposure and chemical damage. Marine-derived exopolysaccharides can also contribute to the cosmeceutical industry as thickening or gelling agents [31]. Marine bacterial EPS have been identified as novel thickening agents, which have the potential to be used as cosmeceutical ingredients. 

## 6. Conclusions and Future Perspectives

Natural products represent the future of cosmetic and cosmeceutical industry. From this perspective, the biological properties of marine natural products have received increased attention. A wide variety of marine molecules, including those deriving from micro- and macro-algae and by-products of the fishing industry, are already on the cosmetic and cosmeceutical market. Conversely, molecules produced by marine bacteria and fungi with potential for these applications are still far from being fully exploited. In the present review, we have highlighted the alternative biomolecules produced by marine bacteria, fungi and fungi-like protists (thraustochytrids) and their advantages compared to other compounds commonly used in cosmetic products. For example, mycosporines and mycosporine-like amino acids produced by marine and marine fungi and bacteria are potentially very efficient natural UV-filters, with strong anti-oxidant activity. Even PUFA and carotenoids produced by marine thraustochytrids might have an important role in cosmetic applications. Similarly, anti-microbial compounds such as chitosan and derivatives extracted from marine fungi and bacteria offer a valid alternative to other synthetic preservatives (e.g., BHA, BHT), being not harmful for skin and environmental health. The natural and biodegradable surfactants extracted from marine microorganisms may reduce the use of synthetic surfactants, thus reducing the impacts on the marine environment. 

The natural products described in this review are extracted from microorganisms inhabiting a wide spectrum of marine habitats. Molecules such as carotenoids, mycosporines and mycosporine-like amino acids are obtained preferentially from organisms subjected to strong light radiation, such as in tropical systems or in shallow water hypersaline pounds. Similarly, compounds with strong anti-oxidant potential are mainly obtained from microorganisms inhabiting extreme systems, such as hydrothermal vents. So far, most of these molecules have been identified in the more accessible and better explored portion of the oceans, such as shallow water ecosystems. However more than 95% of this realm, mostly represented by deep sea, is still uncharted. Therefore, the oceans still have enormous potential for the discovery and development of new compounds and bioactive molecules of microbial origin for technological and cosmetic purposes [2]. The fast discovery rate of previously unknown deep-sea habitats hints at a bright future for identifying new, sustainable and eco-friendly microbial molecules for human well-being.

## Figures and Tables

**Table 1:** Main categories of cosmetic products containing bioactive compounds extracted from microorganisms (bacteria, fungi and thraustochytrids) from different marine habitats.

Main Categories	Bioactive Compounds	Action	Source Microorganisms	Habitat	References
**Photoprotective Compounds**					
Mycosporine	Mycosporine–glutaminol–glucoside and mycosporine–glutamicol–glucoside	UV screening	Marine fungi *Phaeotheca triangularis, Trimmatostroma salinum, Hortaea werneckii, Aureobasidium pullulans* and *Cryptococcus liquefaciens*	Hypersaline water and polar glacial ice	[26]
Mycosporine—like amino acids	Shinorine, porphyra- 334 and novel MAA (mycosporine-glycine-alanine)	UV screening	Marine bacteria *Pseudonocardia* sp. strain P1, Micrococcus p. AK-334, *Actinosynnema mirum* DSM 43827	Oceans, coastal systems,deep-sea, hypersaline, Arctic and Antarctic waters and others	[27]
Carotenoids	β-carotene, astaxanthin, zeaxanthin, cantaxanthin, phoenicoxanthin and echinenone	Skin photo-protection and inhibition of adverse processes induced or mediated by solar UV radiation	Marine bacteria genera *Paracoccus* and *Agrobacterium*; marine fungi genera *Rhodotorula, Phaffia, Xanthophyllomyces*	Marine coastal systems	[28,29,30,31,32]
			*Thraustochytrids*, such as *Thraustochytrium strains* ONC-T18 and CHN-1, *Thraustochytriidae* sp. AS4-A1 (Ulkenia sp.) and *Aurantiochytrium* sp. KH105	Seawater and sediments from tropical and temperate to polar ecosystems, in particular organically enriched systems (e.g., estuaries, leaves of mangrove forests)	[33,34,35]
Benzodiazepine alkaloids	circumdatins I, C, G	UV-A screening activity	Marine fungus of the genus *Exophiala*	Isolated from the surface of the marine sponge *Halichondria panicea*	[36]
**Anti-Aging Products**					
Polysaccharides	EPS	Emulsifying, thickening, absorption and gel formation and anti-wrinkles	Marine fungi and bacteria such as *Agrobacterium* sp., *Alcaligenes faecalis, Xanthomonas campestris, Bacillus* sp., *Zymonas mobilis, Eduarsiella tarda* and *Aureobasidium pullulans, Alteromonas macleodii , Pseudoalteromonas* sp.	Different marine environments, including extreme ecosystems. *Pseudoalteromonas* sp. isolated from antarctic waters	[16,37,38,39,40,41,42,43,44]
	HE 800	Structurally analogous to hyaluronic acid	*Vibrio diabolicus*	Deep-sea hydrotermal vents	[41]
PUFAs	DHA, EPA and omega-3 fatty acids	Soft tissue repair, skin nourishment and stimulation of collagen production	Marine fungi (i.e., *Trichoderma* sp., *Rhodotorula mucilaginosa* AMCQ8A), bacteria (i.e., *Moritella dasanensis, Vibrio* sp., *Pseudomonas* sp. *Shewanella* sp. and *Colwellia* sp.) and thraustochytrids (in particular *Schizochytrium, Aurantiochytrium* and *Ulkenia*)	Thraustochytrids isolated from seawater and sediments from tropical and temperate to polar ecosystems, in particular organically enriched systems (e.g., estuaries, leaves of mangrove forests); bacteria and fungi isolated from coastal to deep-sea habitats	[33,45,46,47,48,49,50,51,52,53,54,55,56]
**Antioxidant Compounds**					
MAAs		Antioxidant activity, scavenging activity of superoxide anions and inhibition of lipid peroxidation	Marine fungi and bacteria	Coastal and open-ocean systems, deep-sea, hypersaline, Arctic and Antarctic ecosystems and others	[26,27,57,58,59]
Carotenoids	Astaxanthin	Antioxidant activity	Marine fungi bacteria and thraustochytrids	Coastal and open-ocean systems, deep-sea, hypersaline, Arctic and Antarctic ecosystems and others	[32,60]
	Saproxanthin and myxol	Reinforce biological membranes, decreasing permeability to oxygen and enhancing protection against oxidation	Marine bacteria family *Flavobacteriaceae*	Antartic marine habitats	[61]
Phenols	Hydroquinone derivatives (e.g., wentiquinone, ethyl 4-(3,4-dihydroxybenzamido)-butanoate)	anti-oxidant activity	Marine fungi *Acremonium* sp. and *Aspergillus wentii N48*	Coastal systems, isolated from brown algae	[62,63]
Isobenzofuranone derivative	4,5,6-trihydroxy-7-methylphthalide	Radical scavenging activity	Marine fungus, *Epicoccum* sp.	Coastal systems, isolated from brown algae *Fucus vesiculosus*	[64]
Exopolysaccharides	EPS2	Radical scavenging activity	Marine fungus *Keissleriella* sp. YS 4108	Marine sediments	[65]
Diketopiperazine alkaloids	Golmaenone and related alkaloids	Radical scavenging activity and UV-A screening function	Marine fungus *Aspergillus* sp.	Isolated from the surface of the marine red alga *Lomentaria catenata*	[66]
Dioxopiperazine alkaloids	Dihydroxyisoechinulin A and related echinulin	Radical scavenging activity and UV-A screening function	Marine fungus *Aspergillus* sp.	Isolated from the surface of the marine red alga *Lomentaria catenata*	[67]
**Skin Whitening Products**					
Pyrone	5-Hydroxy-2-(hydroxymethyl)-4H-pyran-4-one (kojic acid) and derivates (kojic acid dimethyl ether and kojic acid monomethyl ether)	Inhibition of tyrosinase	Marine fungi (i.e., *Aspergillus, Penicillium* and *Alternaria* species)	Different marine ecosystems. A*lternaria* sp. isolated from marine green algae	[68,69]
	α-Pyrone derivate (6-[(E)-Hept-1-enyl]-α-pyrone)	Inhibition of tyrosinase	Marine fungus *Botrytis* sp.	Isolated from the surface of the marine red alga *Hyalosiphonia caespitose*	[70]
	Phomaligol A	Inhibition of tyrosinase	Marine fungus *Alternaria* sp.	Isolated from marine green algae	[69]
	6-*n*-pentyl-α-pyrone and myrothenone A	Inhibition of tyrosinase	Marine-derivated fungus, genus *Myrothecium*	Isolated from the surface of the marine green algae *Entemorpha compressa*	[71]
N-acyl dehydrotyrosine derivatives	Thalassotalic acids A, B and C	Inhibition of tyrosinase	Marine Gram-negative bacterium, *Thalassotalea* sp. PP2-459	Isolated from a marine bivalve	[72]
Compound similar to the structure of homothallin II		Inhibition of tyrosinase	Marine fungus *T. viridae* strain H1-7	Isolated from marine sediments	[73]
Seven different compounds		Inhibition of melanin	Marine fungus *T. viridae* strain H1-7	Isolated from marine sediments	[73]
Dicarboxylic acid	1,7-heptanedicarboxylic acid (azelaic acid)	Inhibition of tyrosinase	Marine fungus *Malasseziales*	Almost every habitat in the marine environment	[74,75,76]
Sesquiterpenes	1β,5α,6α,14-tetraacetoxy-9α-benzoyloxy-7β H-eudesman-2β, 11-diol and 4α,5α-diacetoxy-9α-benzoyloxy-7βH-eudesman-1β, 2β,11, 14-tetraol	Inhibition of tyrosinase	Marine fungus *Pestalotiopsis* sp. Z233.	Isolated from algae *Sargassum horneri*	[77]
Alkyl halides	Methylene chloride	Inhibition of tyrosinase	Marine bacteria genus *Pseudomonas*	Marine sediments	[78]
Anthraquinones	Chrysophanol	Inhibition of tyrosinase	Marine fungus, *Microsporum* sp.	Isolated from the red alga *Lomentaria catenata*	US patent 20140056834A1
Carotenoids	Astaxanthin	Depigmentation properties	Marine bacteria and fungi	Seawater, sediments and marine organisms	[32]
**Antimicrobial Products**					
Polysaccharides	Chitin, chitosan and their derivatives	Antimicrobial activity	Marine fungi such as zygomycetes, chytridiomycetes, ascomycetes, basidiomycetes	Coastal and open-ocean systems, deep-sea, hypersaline, Arctic and Antarctic ecosystems and others	[79,80]
Carotenoids	Astaxanthin	Antimicrobial activity, anti-wrinkle and anti-acne effects	Marine bacteria, fungi and thraustochytrids	Coastal and open-ocean systems, deep-sea, hypersaline, Arctic and Antarctic ecosystems and others	[32]
Parabens	4-hydroxybenzoate alkyl esters	Preventing the growth of yeasts, molds and gram-positive bacteria	The marine bacterial strain, A4B-17, genus Microbulbifer	Isolated from an ascidian	[81]
**Surfactants, Emulsifiers, Thickeners, Stabilizers and Moistourising**					
Polysaccharides	Chitin, chitosan and their derivatives	Moisturising, emulsifying, anti-microbial and adhesive properties, water resistance and absorption promoters	Marine fungi such as zygomycetes, chytridiomycetes, ascomycetes, basidiomycetes	Coastal and open-ocean systems, deep-sea, hypersaline, Arctic and Antarctic ecosystems and others	[82,83,84,85,86,87,88]
Protein polysaccharide complexes, glycolipids, lipopeptides		Dissolving the active compound in other ingredients, emulsifying, skin moisturising and delivery system.	Marine fungi and bacteria such as *Actinobacter, Pseudomonas, Myroides, Streptomyces, Yarrowia, Rhodotorula, Bacillus, Azotobacter, Corynebacterium*	Coastal and open-ocean systems, deep-sea, hypersaline, Arctic and Antarctic ecosystems and others	[89]
